# Immunomodulatory effect of bovine lactoferrin during SARS-CoV-2 infection

**DOI:** 10.3389/fimmu.2024.1456634

**Published:** 2024-10-17

**Authors:** Andrea Marques Vieira da Silva, Thiago Lazari Machado, Ryann de Souza Nascimento, Miguel Pires Medeiros Diniz Rodrigues, Felipe Soares Coelho, Luciana Neves Tubarão, Lorenna Carvalho da Rosa, Camilla Bayma, Vanessa Pimenta Rocha, Ana Beatriz Teixeira Frederico, Jane Silva, Danielle Regina de Almeida de Brito e Cunha, Alessandro Fonseca de Souza, Raphaela Barbosa Gonçalves de Souza, Caroline Augusto Barros, Danielle da Silva Fiscina, Luiz Claudio Pereira Ribeiro, Carlos Alberto Marques de Carvalho, Bruno Jorge Duque da Silva, Rodrigo Muller, Tamiris Azamor, Juliana Gil Melgaço, Rafael Braga Gonçalves, Ana Paula Dinis Ano Bom

**Affiliations:** ^1^ Departamento de Desenvolvimento Experimental e Pré-Clinico (DEDEP), Instituto de Tecnologia em Imunobiológico, Bio-Manguinhos, Fundação Oswaldo Cruz, FIOCRUZ, Rio de Janeiro, Brazil; ^2^ Departamento de Bioquímica, Instituto Biomédico, Universidade Federal do Estado do Rio de Janeiro, Rio de Janeiro, Brazil; ^3^ Laboratório de Pesquisa Multiusuário 04 (LPM-04) Hospital Universitário Graffée Guinle, HUGG/EBSERH, Rio de Janeiro, Brazil; ^4^ Departamento de Patologia, Centro de Ciências Biológicas e da Saúde, Universidade do Estado do Pará, Belém, Brazil

**Keywords:** bovine lactoferrin, COVID-19, immunomodulation, cytokines, TLR4

## Abstract

**Introduction:**

Lactoferrin (Lf) is an important immunomodulator in infections caused by different agents. During SARS-CoV-2 infection, Lf can hinder or prevent virus access to the intracellular environment. Severe cases of COVID-19 are related to increased production of cytokines, accompanied by a weak type 1 interferon response.

**Methods:**

We investigated the influence of bovine Lf (bLf) in the immune response during SARS-CoV-2 infection *in vitro* and *in vivo* assays.

**Results:**

Our results show a strong binding between bLf and TLR4/NF-κB *in silico*, as well as an increase in mRNA expression of these genes in peripheral blood mononuclear cells (PBMCs) treated with bLf. Furthermore, the treatment increased *TLR4/TLR9* mRNA expression in infected K18-hACE2 mouse blood, indicating an activation of innate response. Our results show that, when bLf was added, a reduction in the NK cell population was found, presenting a similar effect on PD-1 in TCD4^+^ and TCD8^+^ cells. In the culture supernatant of PBMCs from healthy participants, bLf decreased IL-6 levels and increased CCL5 in COVID-19 participants. In addition, K18-hACE2 mice infected and treated with bLf presented an increase of serum pro-inflammatory markers (GM-CSF/IL-1β/IL-2) and upregulated mRNA expression of *IL1B* and *IL6* in the lung tissue. Furthermore, bLf treatment was able to restore *FTH1* levels in brain tissue.

**Discussion:**

The data indicate that bLf can be part of a therapeutic strategy to promote the immunomodulation effect, leading to homeostasis during COVID-19.

## 1 Introduction

Coronavirus disease 2019 (COVID-19) has spread worldwide as a pandemic, leading to 776 million confirmed cases, including more than 7.1 million deaths (1). Acute respiratory manifestations are the most common features of severe COVID-19 and may have extrapulmonary involvement (2). Manifesting elevated serum levels of different cytokines, which is called a “cytokine storm,” may contribute to the fatal outcome of COVID-19. Cytokine storm prevention and mitigation could be the key to better clinical outcomes for patients with COVID-19. Thus, some drug-based therapies rely on minimizing the effect of the cytokine storm, such as those with tocilizumab, baricitinib, sarilumab, and corticosteroids (3, 4).

Lactoferrin (Lf), also known as lactotransferrin, is a glycoprotein member of the transferrin family produced commonly by exocrine glands (e.g., mammary and lacrimal glands) and granules of neutrophils (5). Lf was discovered in 1939 and isolated from human (hLf) and bovine (bLf) milk in 1960 (6) as a dimeric protein with 691 and 689 amino acids, respectively, and a molecular weight of about 80 kDa (7).

Lf plays multiple functions related to their capacity of reversible binding to transition metals, such as Fe^3+^ with high affinity, and other ions in a lesser degree. The protein also shows numerous antimicrobial effects, including antibacterial, antifungal, antiviral, antiparasitic, anti-inflammatory, and immunomodulatory activities (8–10).

A broad-spectrum antiviral effect was already assigned to Lf against pathogens such as Influenza A, Human Immunodeficiency, Hepatitis B, and Hepatitis C viruses (11). These antiviral properties were mainly due to the inhibition of virus entry into host cells by attachment to viral particles or blocking cellular receptors (12). Also, the immunomodulatory role of this protein arise from limiting tissue damage by regulation of cytokines, chemokines, and cell surface receptors involved in immunological signaling pathways (13).

Also, a recent study demonstrated the *in vitro* efficacy of Lf against severe acute respiratory syndrome coronavirus 2 (SARS-CoV-2) variants of concern by direct inhibition of virus entry and immunomodulatory mechanisms (14). Furthermore, oral administration of Lf in mice leads to type I interferon production, thus playing a role in antiviral defense, by inhibition of protein synthesis, degradation of viral RNA in infected cells, and enhancement of antiviral immune activity (15).

Lf interacts with Toll-like receptors (TLRs) and promotes regulatory effects in the immune system (16–18). TLR4 is a surface receptor classified as a pattern recognition receptor (PRR) that interacts with several infectious and non-infectious agents, leading to the activation of nuclear factor kappa B (NF-κB), and mitogen-activated protein kinases, leading to phosphorylation of intracellular molecules, such as the MyD88 protein, and contributing to T-cell proliferation as well as natural killer (NK) cells, monocyte, neutrophil, and dendritic cell stimulation (9, 19).

A previous study showed that severely ill patients with COVID-19 have a high concentration of pro-inflammatory cytokines, such as interleukin-6 (IL-6), compared to those who are moderately ill (3). In the same way, IL-2, IL-2R, IL-7, IL-10, granulocyte-colony stimulating factor (G-CSF), interferon-γ-inducible protein 10 (IP-10/CXCL10), monocyte chemoattractant protein (MCP-1/CCL2) MCP-3/CCL7, Interleukin-1 receptor antagonist (IL-1ra), Macrophage Inflammatory Protein-1 Alpha (MIP-1α/CCL3), interferon gama (IFN-γ), and tumor necrosis factor (TNF-α) were observed at high plasma concentration in intensive care unit patients. The acute phase of SARS-CoV-2 infection was associated with a marked leukopenia in up to 80% of hospitalized patients, associated with a dramatic decrease of CD4^+^ and CD8^+^ T cells (20).

Imbalances in the number of mononuclear cells in peripheral blood are related to the inflammatory phase of COVID-19, and some studies have associated these changes with the disease outcome (21–23). Restoration of immune cell numbers and functions has the potential to correct the delicate immune homeostasis required to establish an effective recovery from SARS-CoV-2 infection (24).

In this study, we evaluated the effect of bLf on the cellular immune response and production of pro-inflammatory cytokines using SARS-CoV-2–infected cells derived from patients with COVID-19. In addition, the therapeutic effect of bLf in SARS-CoV-2–infected K18–human ACE2 (hACE2) mice was investigated. Additionally, using *in silico* strategy, we investigated a model of interaction between the Lf molecule and TLR4 in an attempt to provide a possible mechanism of action for this protein.

## 2 Materials and methods

### 2.1 *In vitro* experimental design

#### 2.1.1 Study population

Blood samples were obtained by venipuncture from patients with COVID-19 attended at a public hospital (Gaffrée and Guinle University Hospital) in Rio de Janeiro, Brazil. Fifteen patients were followed up, and blood collection was performed according to COVID-19 symptoms onset at <10 days (T1), 20–30 days (T2), and 40–60 days (T3). SARS-CoV-2 infection was confirmed by real-time reverse transcription polymerase chain reaction (RT-qPCR). All patients signed an informed consent, and the protocol was approved by the Institutional Review Board under certificate number 37079320.4.0000.5258. Participants with negative results for SARS-CoV-2 infection by RT-qPCR and without signals and symptoms were included as a control group and called unexposed (n = 15) (**Table 1**). Demographic data such as age and gender of participants are described in **Table 1**.

**Table 1:** Demographic features of enrolled participants.

	Unexposed (n = 15)	COVID-19 (n = 15)
Age (mean ± SD)*	36 ± 9.4	30.9 ± 10.3
Sex (F/M)	11/4	12/3

*Years; F, female; M, male.

#### 2.1.2 *In vitro* bLf treatment assay

Peripheral blood mononuclear cells (PBMCs) from each participant were isolated and purified by density gradient centrifugation (Ficoll–Paque; Sigma), resuspended in freezing solution CryoStor (STEMCELL Technologies), and cryo-preserved in liquid nitrogen. PBMCs collected at T1, T2, and T3 were cultured in using Culture media for human cells (RPMI) 1640 media (Invitrogen™) supplemented with 1 M 4-(2-hydroxyethyl)-1-piperazineëthanesulfonic acid (HEPES) buffer, 2 mM L-glutamine, 5 μM β-mercaptoethanol, 1 mM sodium pyruvate, 1% non-essential amino-acid solution, 1% (v/v) vitamin, and 10% fetal bovine serum (Invitrogen™) for 18 h (resting) before antigen stimulation. The cells were then incubated for 24 h at 37°C and 5% CO_2_ with recombinant IL-2 (50 ng/mL; Mabtech, #3851-2A) as a positive control. Afterward, PBMC samples from healthy participants unexposed to COVID-19 and from patients with convalescent COVID-19 (T2) were treated with different concentrations of apo-bLf (CAS BioSciences, New York, NY, USA) at 1, 5, and 10 mg/mL, and the cells were then incubated for 24 h at 37°C and 5% CO_2_, after resting of 18 h, whereas PBMC samples from T1 were treated with bLf at 10 mg/mL, after resting. The cells were incubated for 48 h at 37°C and 5% CO_2_. Subsequently, the cells were collected for RNA extraction using the TRIzol reagent (Invitrogen™), according to the manufacturer’s instructions.

#### 2.1.3 Immunophenotyping by flow cytometry

After initial incubation, cells were harvested, and the pellet was washed with Fluorescence Activated Cell Sorting (FACS) buffer (25). After that, the cells were resuspended in live-dead blue dye solution, incubated for 15 min, washed with FACS buffer, and centrifuged at 400 g for 10 min. Then, the cells were incubated for 25 min at 2°C–8°C with a mix of human antibodies: CD8 Brilliant Violet 605 (clone SK1), CD56 PECy5 (clone B159), CD3 FITC (clone UCHT-1), CD16 PECF594 (clone 3G8), CD4 APC (clone L200), CD69 Brilliant Violet 421 (clone FN50), and CD279 (PD-1) Brilliant Violet 711 (clone EH12.1), all purchased from BD Biosciences (San Diego, CA, USA). The samples were washed with FACS buffer, centrifuged at 400 g for 10 min, homogenized with 1% paraformaldehyde solution, and analyzed using LSR Fortessa (BD Biosciences) followed by offline analysis by FlowJo software (BD Biosciences).

#### 2.1.4 Cytokine detection in PBMC supernatant

To verify the levels of IL-6, C-X-C motif chemokine ligand 8 (IL-8/CXCL8), C-C motif chemokine ligand 5 (rantes/CCL5) cytokines in the supernatant of stimulated PBMC, an *in-house* multiplex liquid microarray test was performed. To this end, 10^6^ xMAP microspheres (Luminex Corporation, Austin, Texas, USA) were coupled with anti-human mouse purified monoclonal antibodies at the following concentrations: anti–IL-6 at 100 μg/mL, anti-CXCL8 at 50 μg/mL, and anti-CCL5 at 100 μg/mL (all from Abcam Plc, Cambridge, UK). Coupling reactions were performed using the Amine Coupling Kit (Bio-Rad) following the manufacturer’s instructions. For quantitation assay, the Bio-Plex Pro Human Cytokine Standard 27-plex, Group I (Bio-Rad, Hercules, CA, USA), was used as a standard curve following the manufacturer’s instructions. Supernatant samples from cell culture were diluted 1:10, and standards were incubated in duplicates with coupled microspheres at 600 rotations per Minute (rpm) for 30 min at 37°C. The microspheres were washed three times with wash solution [Phosphate buffered saline (PBS) (pH 7.4) + 1% bovine serum albumin + 0.02% Tween 20 + 0.005% sodium azide] and incubated with anti-human goat polyclonal biotinylated antibodies (0.1 μg/mL) against all the analyzed cytokines (R&D Systems, Minneapolis, MN, USA) at 600 rpm for 30 min at 37°C. Subsequently, the microspheres were washed, incubated with streptavidin-Phycoerythrin (PE) (BD Biosciences) at 600 for 10 min at 37°C, and resuspended in the wash solution. The median fluorescence intensity (MFI) of each reaction was quantified in pg/mL using the Luminex MagPix system (Luminex Corporation, Austin, Texas, USA). Cytokine concentrations were calculated by interpolating MFI of samples with the standard curve and a four-parameter analysis was applied using the SoftMax Pro v5.4 software (Molecular Devices, San Jose, CA, USA).

#### 2.1.5 Cytokines gene expression in bLf-treated PBMC

Complementary DNA (cDNA) was synthesized from 500 ng of total RNA using the High-Capacity cDNA Reverse Transcription Kit (Thermo Fisher Scientific). Thirty-two transcripts were quantified, using *B2M*, *GAPDH*, *18S*, and *PPIA* genes as endogenous controls of expression (genes and primer descriptions are given in **Supplementary Table S1**). Analysis of gene expression was performed using Fluidigm (Biomark platform) assays, as described elsewhere (26), and according to the manufacturer’s instructions. Data were normalized by division of the cycle threshold (Ct) mean value of targets by Ct mean of reference genes. Results were expressed on a logarithmic scale.

### 2.2 *In silico* molecular docking to evaluate the protein–protein interactions of bLf with TLR4 and NF-κB

The *in silico* experiments were performed using Cluspro 2.0 server (https://cluspro.bu.edu/login.php) to molecular docking the protein–protein interaction model for bLf (Protein Data Bank (PDB): 1BLF) with TLR4 from *Homo sapiens* (human samples) (PDB: 4G8A) and bLf with NF-κB from *Homo sapiens* (human samples) (PDB: 1SVC). The top score models posed with the lowest energy were chosen to be analyzed through the PDBsum server (http://www.ebi.ac.uk/pdbsum) to get the residue interactions and bounding information. The PyMoL software (http://www.pymol.org/pymol) was used to calculate the root mean square deviation (RMSD) by superimposition between the ligand’s atoms and the visualization of the protein–protein docking generated.

### 2.3 *In vivo* experimental design

#### 2.3.1 SARS-CoV-2 infection and bLf treatment of K18-hACE2

**Figure 1:**
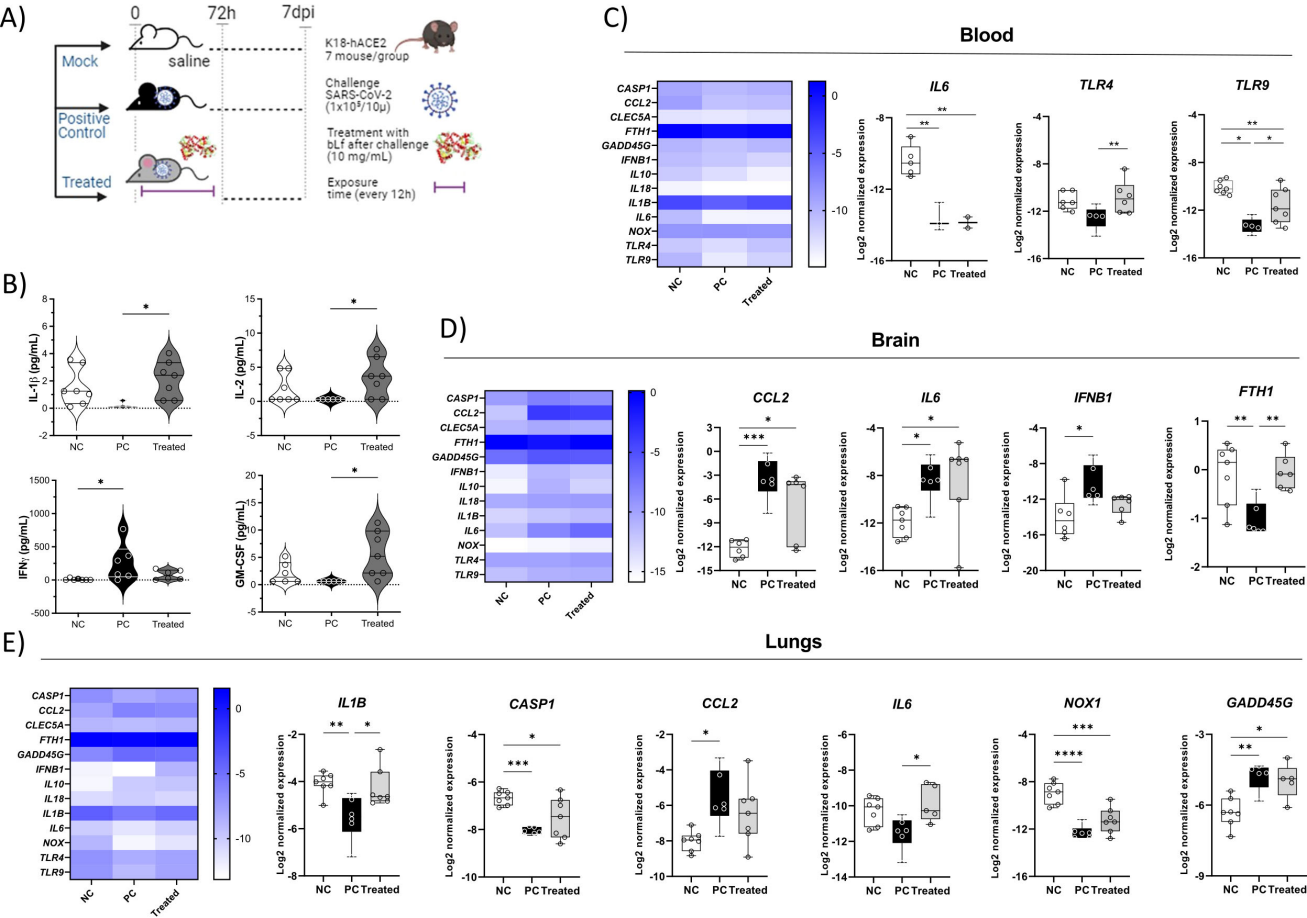
Cytokine levels in different tissues, showing the immunomodulatory effect of bLf in K18-hACE2 mice infected with SARS-CoV-2. **(A)** Experimental design; **(B)** serum levels of IFN-γ, IL-1β, GM-CSF, and IL-2; **(C)** *IL6*, *TLR4*, and *TLR9* gene expression in whole blood; **(D)** *CCL2*, *IL6*, *IFNB1*, and *FTH1* gene expression in brain tissue; **(E)** *IL1B*, *CASP1*, *CCL2*, *IL6*, and *NOX1* gene expression in lung tissue. Kruskal–Wallis tests with Dunn’s *post-hoc* tests were applied to compare groups. *p < 0.05, **p < 0.005, ***p < 0.0005, and ****p < 0.00005. NC, negative control; PC, positive control (COVID-19); treated, treated with bLf.

Twenty-one K18-hACE2 transgenic mice of both sexes were used as a SARS-CoV-2 infection model. Animals were 17 to 20 weeks old, weighing 20–25 g, SARS-CoV-2 naïve, and captivity colony born in the Mice Breeding Service of the Institute of Science and Technology in Biomodels of Fiocruz (Rio de Janeiro, Brazil). Of this total, three experimental groups (n = 7) were generated: negative control (NC), subjected to saline administration; a positive control (PC) group, infected 10^5^ SARS-CoV-2 particles (Wuhan strain); and a group that received both the viral particles and the apo-bLf at 10 mg/10 μL (treated) for 72 h (at 12-h intervals). K18-hACE2 mice were inoculated through the intranasal route by trained staff and observed daily to record body weight and clinical signs of illness (bristling fur, arched back, respiratory alteration, eye discharge, and limited mobility). After 7 days post-infection (dpi), mice were euthanized by CO_2_ asphyxiation followed by cervical dislocation (**Figure 1A**). Whole blood as well as pieces of lungs and brain tissues were harvested and stored using a stabilizer solution of Invitrogen™, TRIzol (blood), or RNAlater (lungs and brain), according to the manufacturer’s instructions. This research protocol was approved by the Ethics Committee on the Use of Animals of Fiocruz under certificate number LW-17/20.

#### 2.3.2 Cytokine detection in K18-hACE2 mouse serum

Cytokines in serum samples from K18-hACE2 mice at the seventh dpi were detected using ProcartaPlex Mouse Th1/Th2 Cytokine Panel, 11plex kit (Thermo Fisher Scientific), according to the manufacturer’s instruction. The samples were assayed in duplicate to quantify the levels of the cytokines IL-1β, IL-2, IL-4, IL-5, IL-6, IL-12p70, IL-13, IL-18, IFN-γ, TNF-α, and Granulocyte-macrophage colony-stimulating factor (GM-CSF). The results were analyzed as described in Section 2.1.4.

#### 2.3.3 Cytokine/chemokine gene expression in tissues and whole blood

To evaluate the gene expression profile at 7 dpi, tissue mRNA was extracted using the RNeasy Plus Mini Kit (Qiagen), and whole-blood mRNA was extracted by the TRIzol method, both procedures according to manufacturer’s instructions, followed by cDNA synthesis from 500 ng of total RNA using the High-Capacity cDNA Reverse Transcription Kit (Applied Biosystems). The cytokines*/chemokine IL1B*, *IL6*, *IL18*, *FTH1*, *NOX1*, *IL10*, *CASP1*, *GADD45G*, *IFNB1*, *TLR4*, *TLR9*, *CCL2*, and *CLEC5A* transcripts were quantified, using *B2M*, *GAPDH*, and *PPIA* genes as endogenous controls of expression (gene and primer descriptions are given in **Supplementary Table S1**); qPCR was performed using SYBR^®^ Green master mix (Applied Biosystems), 400 nM each primer (forward and reverse) and 5 ng each cDNA. The cycling conditions used were 10 min at 95°C, 15 s at 95°C, and 4 min at 60°C, for 45 cycles for DNA denaturation and amplification carried out using the QuantStudio Pro 7 Real-Time PCR System (Applied Biosystems). Using the LinRegPCR software, the mean PCR efficiency per amplicon was determined and used to calculate the start concentration per sample (N0), expressed as arbitrary fluorescence units. Data were normalized by the division of N0 mean of targets by N0 mean of the reference genes. Results were expressed on a logarithmic scale.

### 2.4 Statistical analysis

Kolmogorov–Smirnov test was performed to identify the normality assumption of the data. The comparisons between the explored groups were performed using the Mann–Whitney U test. For the *ex vivo* data, the comparison was performed among unexposed subjects and the different time points of blood collection were made for the COVID-19 group. For the *in vitro* data from FACS, the comparison was performed between unstimulated (mock) and all concentrations of bLf used in the cell culture assay. Bar graphs represent mean ± standard deviation (SD). Cell frequencies above 1% were considered for the final analysis of the flow cytometry data, and differences were considered statistically significant when p ≤ 0.05. The cytokines detected were analyzed by comparing the SARS-CoV-2–infected and bLf-treated groups, using the Kruskal–Wallis test with Dunn’s post-test, and differences were considered statistically significant when p ≤ 0.05. Log2-normalized expression was compared among NC, PC, and treated groups using the Kruskal–Wallis test with Dunn’s post-test, and differences were considered statistically significant when p ≤ 0.1. Graphs and statistical analysis were performed using Prism software, v8.4.2 (San Diego, CA, USA).

## 3 Results

### 3.1 The relative amount of circulating NK and T cells affected by SARS-CoV-2 infection

**Figure 2:**
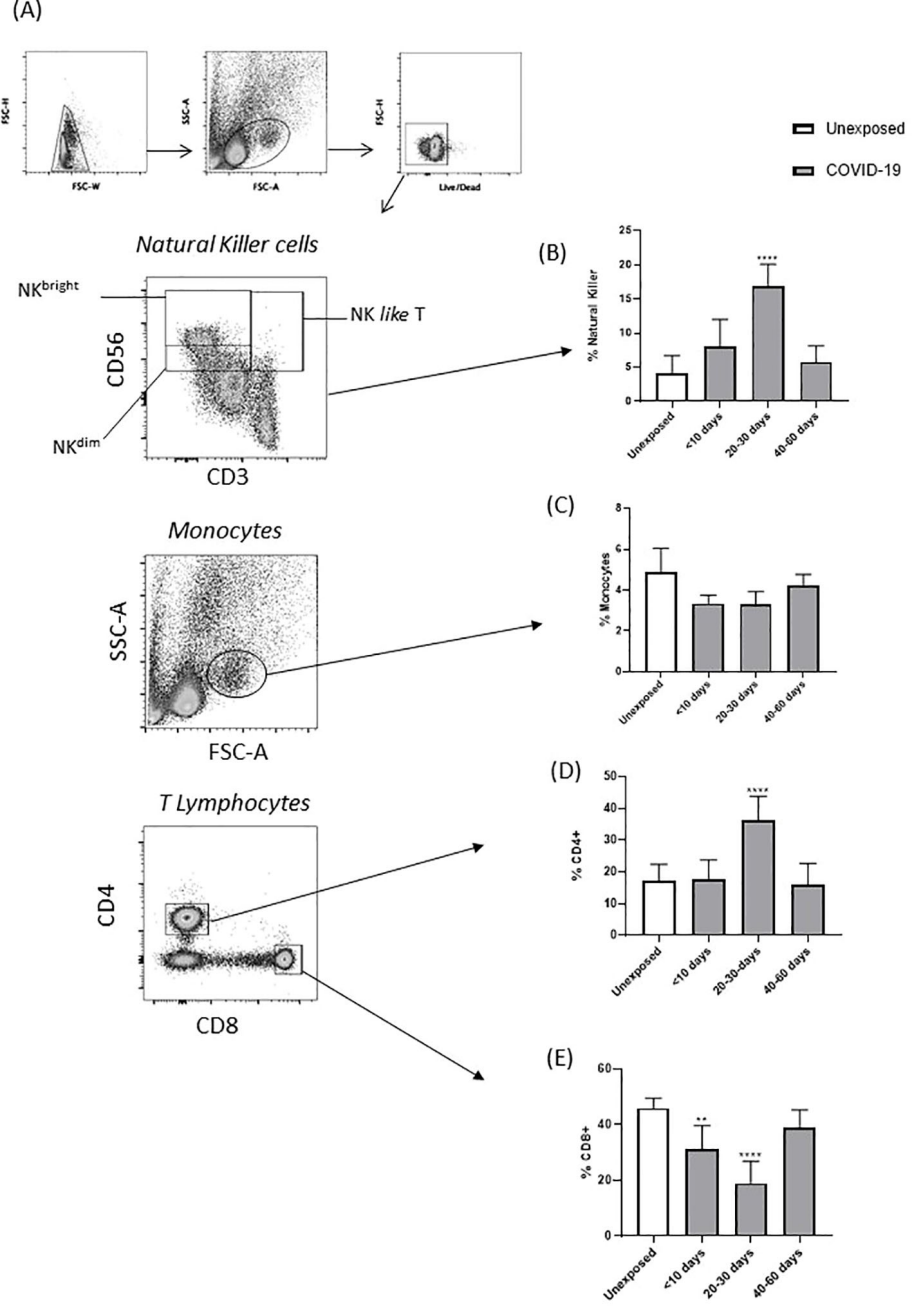
Percentage of circulating immune cells from patients with COVID-19 and unexposed individuals, at different stages of the disease. **(A)** Gate strategy accessing the subpopulation of PBMCs by *ex vivo* immunophenotyping. **(B)** Percentage of total NK cells, **(C)** total monocytes, **(D)** total CD4^+^ T cells, and **(E)** total CD8^+^ T cells, obtained from unexposed individuals and patient with COVID-19. Percentage of obtained from unexposed individuals and patients with COVID-19 (**p < 0.01, and ****p < 0.0001).

Comparisons of blood from healthy subjects (unexposed) and patients with COVID-19 revealed that the percentage of circulating NK (CD56^+^) and CD4^+^ T cells significantly increased at 20–30 days after the onset of symptoms (**Figures 2A, B, D**). In contrast, CD8^+^ T cells were reduced at the same time point (**Figures 2A, E**). Total monocytes (CD14^+^) were not affected in the follow-up, and a restoration in CD8^+^ T cells was observed 40–60 days after symptom onset (**Figures 2A, C**).

### 3.2 *In vitro* effects of bLf on NK and T cells and secreted cytokines in the convalescent phase

**Figure 3:**
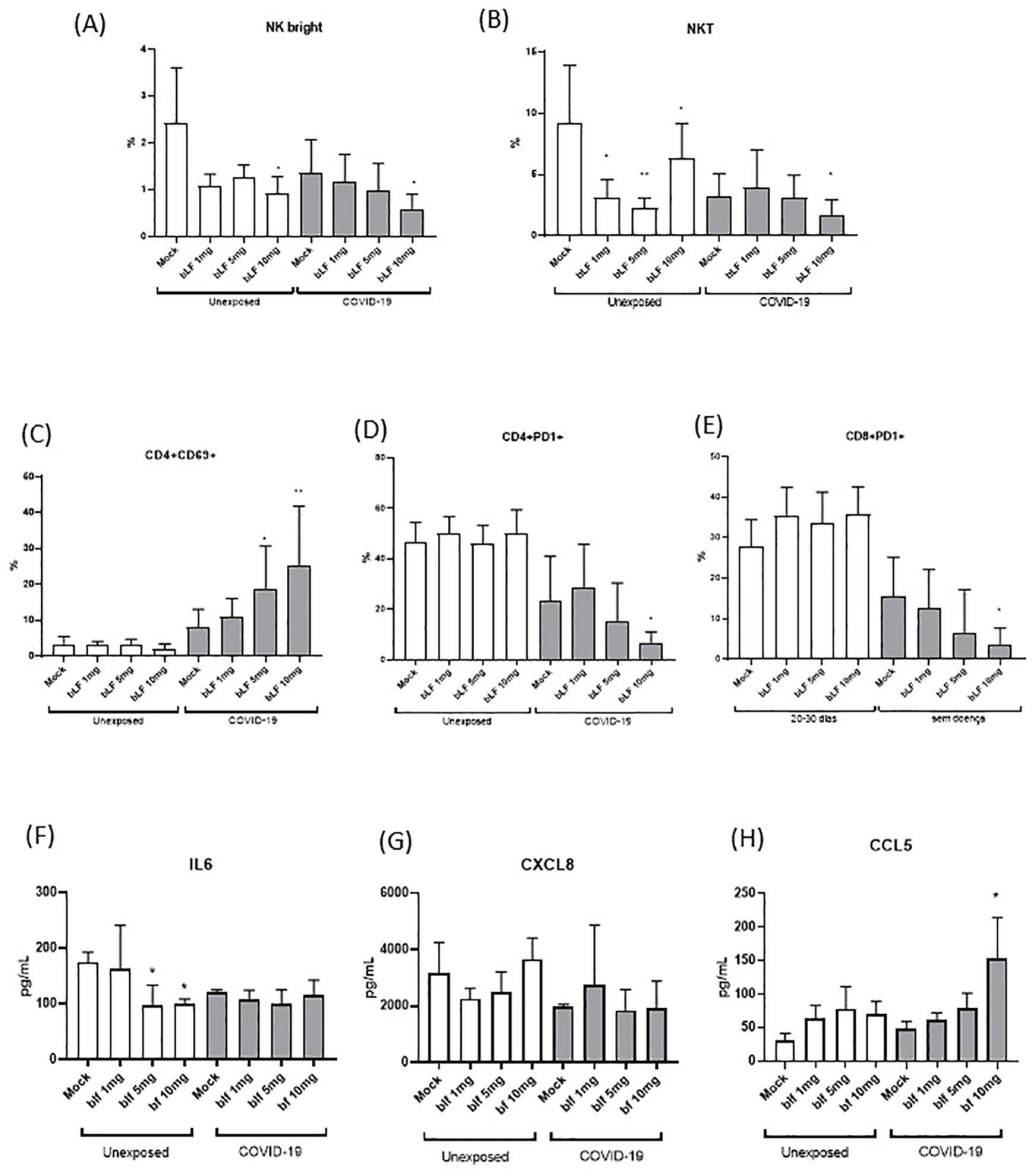
*In vitro* effects of bLf on subpopulations of NK and T cells from unexposed and COVID-19 groups. **(A)** Percentage of NK^bright^ (CD3^−^CD56^high^CD16^−^), **(B)** NKT(CD3^+^CD56^+^), **(C)** CD4^+^ activated T cells (CD69^+^), **(D)** exhausted CD4^+^ T cells (PD-1^+^), and **(E)** exhausted CD8^+^ T (PD-1^+^) cells upon stimulation with different bLf concentrations (1, 5, and 10 mg/mL), as well as unstimulated (mock). Levels of **(F)** IL-6, **(G)** CXCL8 (IL-8), and **(H)** CCL5 (RANTES) detected in supernatant obtained from cell stimulation conditions. *p < 0.05 and **p < 0.01.

Considering the results for the COVID-19 group at 20–30 days after the onset of symptoms, PBMCs from this time point were chosen for *in vitro* cultivation with bLf to explore their effects on the subpopulations of NK, CD4^+^ T, and CD8^+^ T cells. Although we found that patients with COVID-19 showed an increase in the total NK population, in the subpopulation of NK cells, NK^bright^ cells (CD3^−^CD56^high^) and NK-like T cells (NKT) were showed a decrease in the COVID-19 exposed group (**Figures 3A, B**). Furthermore, bLf (10 mg/mL) reduced the subpopulation of NK^bright^ cells (CD3^−^CD56^high^) in individuals exposed and unexposed to COVID-19 (**Figure 3A**). The NKT subpopulation showed an apparent reduction in concentrations of 1, 5, and 10 mg/mL bLf in unexposed individuals, whereas those exposed only showed a reduction in bLf at a concentration of 10 mg/mL (**Figure 3B**). When T cells were evaluated under bLf treatment, the activation marker (CD69) was found to be raised for CD4^+^ T cells at 5 and 10 mg/mL bLf (**Figure 3C**). In addition, the programmed death marker (CD279/PD-1) expression was diminished in bLf at 10 mg/mL for CD4^+^ and CD8^+^ T cells, but only in the COVID-19 group (**Figures 3D, E**). Secreted IL-6 levels were diminished upon bLf treatment at 5 and 10 mg/mL in unexposed PBMC culture (**Figure 3F**). Although CXCL8 levels were unaltered in both groups (**Figure 3G**), high levels of CCL5 were detected after bLf treatment at 10 mg/mL in the COVID-19 group (**Figure 3H**).

### 3.3 Increased expression of NFKB, IFIT1, NCF4, and TLR4 genes induced by bLf in an acute phase

**Figure 4:**
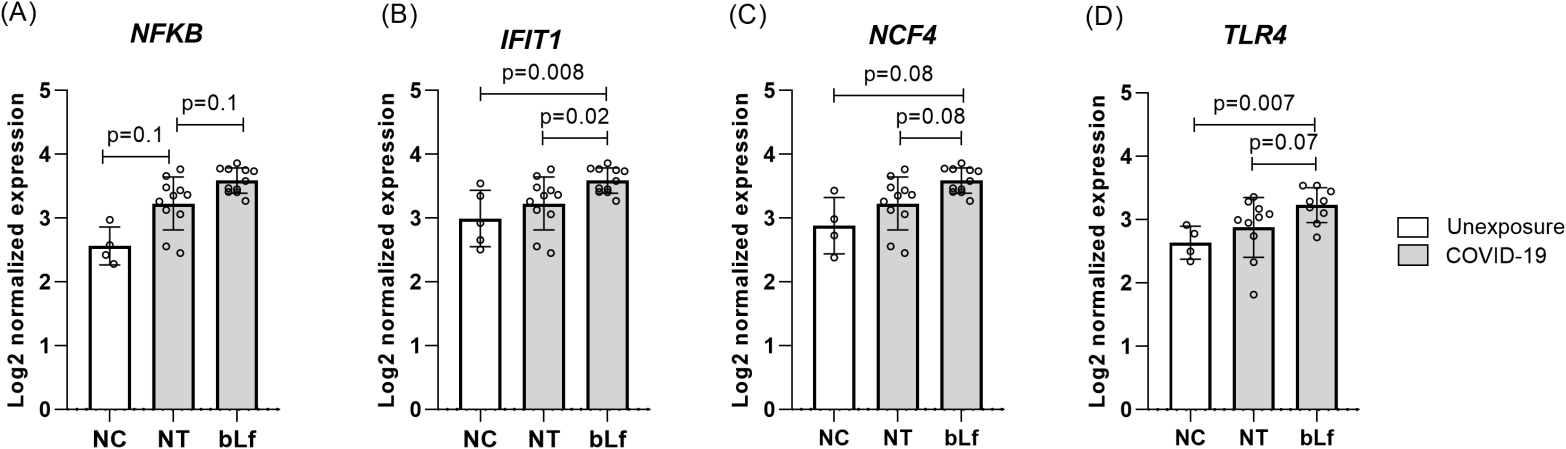
Upregulated genes expression after *in vitro* bLf treatment of PBMCs in the acute phase of COVID-19. Expression levels of *NFKB* **(A)**, *IFIT1* **(B)**, *NCF4* **(C)**, and *TLR4* **(D)**. Kruskal–Wallis tests with Dunn’s *post-hoc* tests were applied to compare groups. NC, negative control (unexposed); NT, no treatment (COVID-19); bLf, treatment with bLf.

Based on the results obtained in the immunophenotyping, treatment with bLf at 10 mg/mL had a greater effect on the frequency of cell subpopulations. Thus, the PBMCs of the acute phase COVID-19 group (T1) were treated with this concentration for evaluation of different cytokines by mRNA expression. Evaluating only the effect of Lf on PBMCs from acute participants, mRNA expression of *NFKB* (nuclear factor kappa B), *IFIT1* (ferritin heavy chain 1), *NCF4* (neutrophil cytosolic factor 4), and *TLR4* (TLR4) were upregulated with statistically significant differences compared to those in the NC and no treated (NT) groups as shown in **Figure 4**. However, no significant difference was seen in pro-inflammatory and regulatory cytokine genes.

### 3.4 Molecular docking shows interactions of bLf with TLR4 and NF-κB

**Figure 5:**
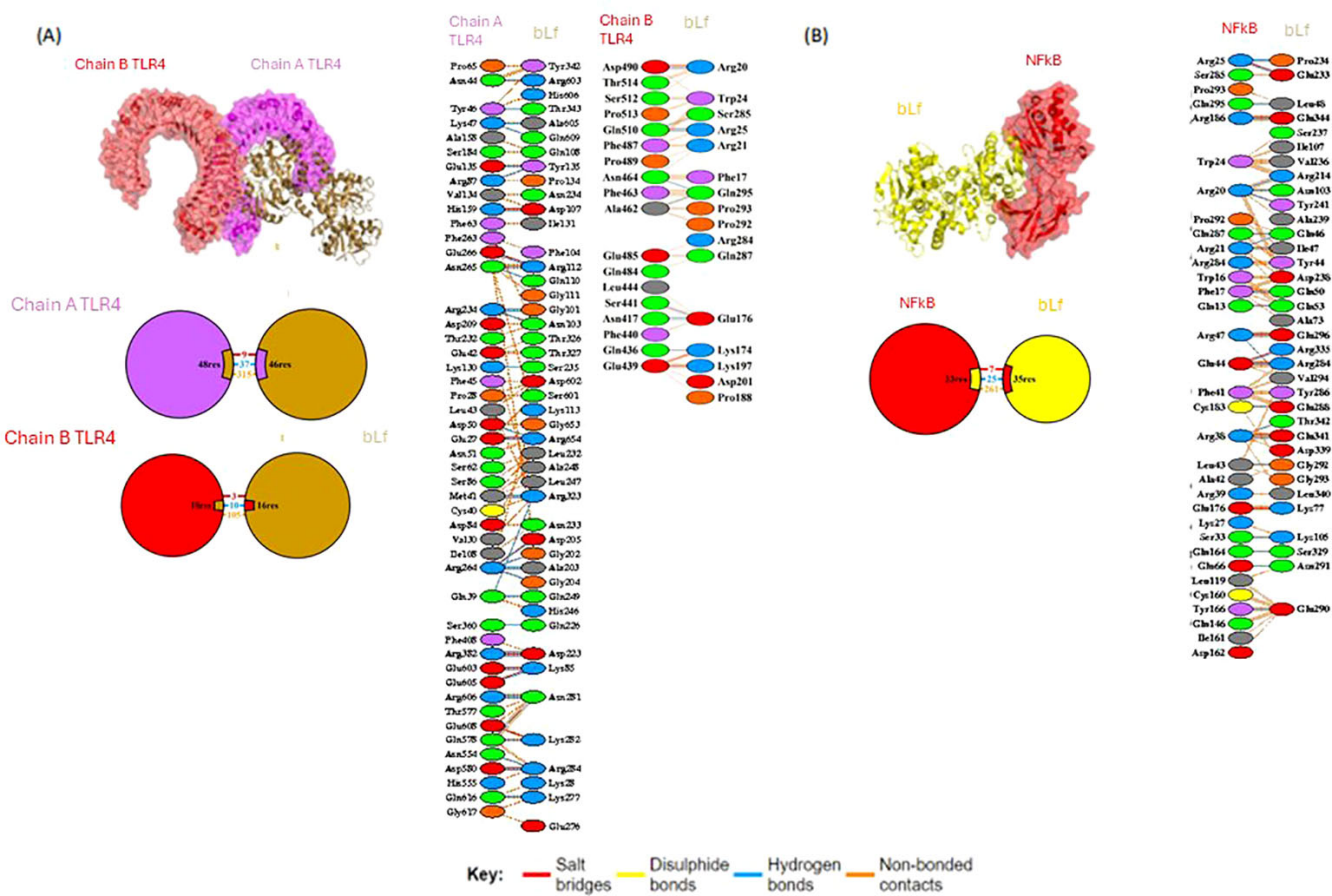
Molecular docking of bLf with TLR4 and NF-κB. Structure of molecular docking pose of bLf with TLR4 **(A)** and NF-κB **(B)**, showing residue interactions as well as bound types.

Using *in silico* analysis, 66 residues (48 from chain A and 18 from chain B) of TLR4 (PDB: 4G8A) were found interacting with 62 residues of bLf (PDB: 1BLF). The binding information is summarized in **Figure 5** and **Table 2**. In addition, bLf also interacted with NF-κB (PDB: 1SVC) through 35 and 33 residues, respectively. Both TLR4 and NF-κB had high weighted scores with low energy and good RMSD values (<0.5 Å) in their interactions with bLf (**Table 2**). Such strong interchain interactions of bLf with a cell surface receptor (TLR4) and an intracellular protein (i.e., NF-κB) may simulate the internalization of the molecule, suggesting a potential activation of immune cells.

**Table 2:** Description of the top models of TLR4/bLf and NF-κB/bLf docking complexes as assessed by the ClusPro 2.0 method.

	Weighted score	Lowest energy
ClusPro 2.0 model	TLR4 - bLf	NF-κB - bLf
Balanced (kcal/mol)	−1,210.1	−911.1
Electrostatic favored (kcal/mol)	−1,045.8	−1072.3
Hydrophobic favored (kcal/mol)	−998.8	−956.0
van de Waals + electrostatic (kcal/mol)	−333.2	−264.1
RMSD (Å)	0.262	0.368

RMSD, root mean square deviation value.

### 3.5 Immunomodulatory effect of bLf on SARS-CoV-2 acute infection *in vivo*

During the monitoring of K18-hACE2 mice, a lower percentage of weight loss was observed in the animals that received bLf (treated) in comparison to that in PC group, whereas the NC group did not show any weight loss (**Supplementary Figure S1**). There was also a slight decrease in clinical signs in animals from the treated group, with no significant difference in the infected group (PC) (**Supplementary Figure S2**). These clinical aspects suggest that bLf exerts a favorable effect in terms of COVID-19 outcome. Analyzing SARS-CoV-2 RT-qPCR, it was not possible to observe significant changes in the viral load of treated mice and PC group, in samples of the nasal swabs from 7 days post-challenge (data not showed). Hence, we hypothesized that the immunomodulatory properties of bLf could be protective against COVID-19 in our mouse model.

The immunomodulatory effect of bLf on COVID-19 reflected in a differential cytokine profile in serum, blood cells, and lung/brain tissues of treated mice. Among cytokines analyzed in the serum, IFN-γ levels were lower in the treated bLf group and the NC group compared to those in the untreated mouse group PC (p < 0.05). The pro-inflammatory cytokines IL-1β, IL-2, and GM-CSF, but not IFN-γ, showed higher levels in treated animals than that in the PC group (**Figure 1B**). In the blood, the treated group also presented a high expression of *TLR4* and *TLR9* when compared to the PC group (**Figure 1C**). In the brain, the virus led to high expression of *CCL2*, *IL6*, and *IFNB1* and low *FTH1.* The bLf treatment led to increase *FTH1* expression (**Figure 1D**). Analyzing the lung gene expression levels of the PC group in comparison to that of the NC group, it was observed that the levels of *CCL2* and *GADD45G* increased and that of *IL1B*, *CASP1*, and *NOX1* increased. The bLf treatment induced increased levels of *IL1B* and *IL6* (**Figure 1E**). Thus, these data suggest that bLf treatment was able to increase the systemic and lung levels of pro-inflammatory cytokines and the homeostatic effects in iron metabolism in the treated group, contributing to an early and beneficial inflammatory response.

## 4 Discussion

The COVID-19 pandemic is still a threat, due to the fast virus spread of new variants (27, 28). The main feature of COVID-19 pathogenesis is the dysregulation of immunological responses called cytokine storm. Despite the development of effective vaccines, there is no specific treatment available, and some subjects remain vulnerable to severe manifestations, such as pregnant women, elderly, and immunocompromised people. Hence, it is imperative to develop a therapeutic strategy capable of being used in those risk groups. Here, we utilized cells from patients with COVID-19, *in silico* and *in vivo* approaches, for investigating the potential immunomodulatory effect of bLf in COVID-19 infections. Our findings show that bLf can bind to TLR4 and NF-κB, modulating the frequency of circulating T and NK subtypes cells, as well as reestablishing homeostatic levels of immunological mediators.

Lf is a pleiotropic molecule that triggers both anti- and pro-inflammatory events, with an important role in general immunity toward the restoration of physiological homeostasis. Some studies demonstrated the anti-inflammatory effect of Lf on immune cells (macrophages, NK cells, and neutrophils) (5, 26). It has been shown that the treatment with Lf inhibits the formation of neutrophil extracellular traps in neutrophils exposed *in vitro* to polyclonal stimuli such as phorbol 12-myristate 13-acetate (5). Moreover, Lf presents a protective role upon exposition to exogenous antigens, serving as a mediator for the activation and migration of macrophages/monocytes and dendritic cells (13, 29, 30). Here, the reduction of IL-6 levels and the frequency of NK cells in participants’ unexposed (healthy) samples with bLf reinforced its potential immunomodulatory effect.

In the COVID-19 context, our findings suggest that the treatment with bLf could help develop a responsive immune response in the COVID-19 acute phase. In terms of innate immune responses, peripheral blood cells from our clinical cohort and mice infected with SARS-CoV-2 presented a cytokine storm profile observed in previous studies. Studies demonstrated that such a profile can be favorable during the acute phase by triggering antiviral responses but is associated with severity in the convalescent phase when the immunopathological responses can cause damage (27, 31). Here, bLf treatment in the acute phase led to high levels of pro-inflammatory and antiviral cytokines, including high levels of TLR4, NF-κB, and downstream genes, whose activation events are pivotal for fighting against viral infections.

Cell surface receptors (PRRs and TLR4) bind to several infectious and non-infectious agents triggering the activation of NF-κB, contributing to T-cell and other immune cell proliferation (9, 18, 19, 32). The binding of bLf to TLRs was previously demonstrated with an immunomodulatory effect, facilitating maturation, differentiation, and functional activity of neutrophils, monocytes, and dendritic cells (16). Nevertheless, the molecular interaction of bLf with TLR4 and NF-κB has not been well explored, especially during SARS-CoV-2 infection. Here, our *in vitro* study showed a significant expression of both *TLR*4 and *NFKB* after bLf treatment of immune cells from COVID-19 group, as indicated by RMSD value of 0.262 Å for TLR4 from human samples binding to bLf, whereas Ohto et al. (33) showed an RMSD value of 1.6 Å among TLR4 and putative ligand lipopolysaccharide (LPS). Also, analyzing the residues present in the binding sites, our molecular docking suggests the activation of both TLR4 and NF-κB because of bLf interaction. Mukund et al. (34) showed some binding residues of NF-κB (Lys52, Ser243, Asp274, and Lys275) presenting the inhibition of the translocation to the nucleus and expression of NF-κB in breast cancer cell lines (35). These active site residues did not participate in any interaction observed in our *in silico* data, suggesting that they do not affect the binding between bLf and NF-κB (34). Also, it was observed *in vitro* in cell lineage the participation of carbohydrate chain human Lf in activation of TLR4-mediating innate immunity (36). In this context, Ráscon-Cruz et al. (2021) showed that five potential glycosylation sites of bLf (Asn233, Asn281, Asn368, Asn476, and Asn545) can be exposed on its surface and may participate in the recognition of specific receptors (16). Our *in silico* findings detected two residues, Asn233 and Asn281, binding to TLR4, suggesting that these residues may participate in the immunomodulatory mechanism of bLf observed in the activation of NK and T cells during COVID-19. Likewise, *in vivo* bLf treatment also induced *TLR4* and *TLR9* in K18-hACE2 mouse peripheral blood. Both the pathogen and bLf were inoculated through the intranasal route to simulate viral tropism by the respiratory system. Allegedly, this is the first report to use this model to evaluate bLf immunomodulatory effect against acute COVID-19.

In COVID-19 group, NK cells (CD56^+^) and helper CD4^+^ T cells (Th1 and Th2) were found in the lungs of deceased patients and, with increased frequency in peripheral blood, were related to the inflammatory phase (21–23). In addition, it was demonstrated that SARS-CoV-2 can induce the activation of these immune cells (37–39). On the other hand, lymphopenia was also described by several studies, as well as impairment of IFN production, suggesting an ability of SARS-CoV-2 to perform immune escape or suppress the immune response (35, 40, 41). Similar results were detected in our study with patients in the acute phase of COVID-19, which showed an increase in NK cells and CD4^+^ T cells (20–30 days), but a decrease in CD8^+^ T cells up to 10 days and between 20 and 30 days from the onset of symptoms.

Moreover, bLf was able to reduce the percentage of NK cells subset even for COVID-19 or unexposed (healthy) subjects in the *in vitro* assay. Nevertheless, Kuhara et al. (2006) reported that oral administration of bLf in healthy mice increased NK cell populations and IFN-γ production in the peripheral blood and spleen in a dose-dependent manner, suggesting that bLf enhances NK cell activity (15). In contrast to our *in vivo* data, K18-hACE2 mice treatment with bLf showed lower serum IFN-γ levels on 7 dpi of SARS-CoV-2 infection. Regarding PD-1, Zeng et al. (2020) showed PD-1 overexpression by activated T cells associated with fatal outcomes in critical patients with COVID-19 (22). Here, in T cells expressing PD-1, bLf could impair its frequency, specifically in COVID-19 samples. Meanwhile, an increase of CD4^+^ T cells expressing CD69^+^ together with CCL5 levels was noted in our experiments using bLf on COVID-19 samples. Pérez-García et al. (2022) showed that low CCL5 expression levels in the upper respiratory tract are associated with COVID-19 severity (42). CCL5 chemokine combined with CCR5, CCR3, and CCR1 receptors is upregulated by Th1 responses and downregulated by Th2 responses, with an important role in Th1 cell recruitment and activation (43), which suggests a positive effect of bLf in our experiments. In addition, treated bLf caused an increase in GM-CSF, IL-1β, and IL-2 in serum levels *in vivo*, which returned to the homeostatic level.

That same effect was seen on the mRNA expression of several genes related to pro-inflammatory pathways in brain and lung tissues, confirming the modulating effect of bLf. Also, the high expression of *FTH1* is consistent with findings that demonstrate the role of bLf as a physiological orchestrator of iron and inflammatory homeostasis through its ability to modulate the expression of the major iron proteins, both in *in vitro* and *in vivo* studies as well as in clinical trials (10). Additionally, a higher *FTH1* expression was observed in the brain tissue of the bLf-treated group, indicating that bLf can cross the blood-brain barrier and suggesting that brain cells are important targets for its action (44).

In addition to its well-documented immunomodulatory properties, bLf exhibits a multifaceted antiviral activity against SARS-CoV-2, acting at various stages of the viral lifecycle. Beyond modulating the immune response, bLf has been shown to block the interaction between the viral spike protein and heparan sulfate proteoglycans, which serve as alternative receptors facilitating viral entry (45, 46). Furthermore, bLf inhibits the activity of the serine protease Transmembrane Serine Protease 2 (TMPRSS2), crucial for viral priming, thereby preventing the virus from entering host cells (47). Additionally, bLf has been reported to reduce viral replication by inhibiting the RNA-dependent RNA polymerase (48, 49) and to blunt the main viral protease Mpro (also known as 3CLpro), which is essential for viral protein processing and maturation (50). These diverse mechanisms of action likely contribute to the observed antiviral effects of bLf in our study and highlight its potential as a broad-spectrum antiviral agent against SARS-CoV-2.

Furthermore, the antiviral efficacy of bLF can vary depending on the SARS-CoV-2 variant, as demonstrated by Wotring et al. (14), who found that bLf and its derivative, bovine lactoferricin (bLfcin), were particularly effective against the Alpha (B.1.1.7) variant. In our study, we used the Wuhan strain, and, although this choice provides valuable insights, it is important to consider that different variants, due to their distinct mutations, may respond differently to bLf. These variations could influence the extent of bLf antiviral effects, as certain mutations might alter the virus interaction with host cell receptors or its susceptibility to Lf mechanisms of action. However, a more recent study by Alves et al. (51) revealed that SARS-CoV-2 Wuhan and Omicron (BA.1) strains were equivalent in terms of sensitivity to bLf during infection of Vero cells, regardless of the iron-saturation state of the protein. Further studies are thus needed to assess bLf effectiveness across a broader range of SARS-CoV-2 variants.

As a limitation of the current study, the high concentration of bLf used for the *in vitro* assays concerning the constitutive levels of Lf in humans could exacerbate the immune events induced by this molecule. For *in vivo* experiments, an additional collection point between 0 and 7 dpi would, perhaps, be more informative for the immunomodulatory effect of Lf, presenting a greater number of differentially expressed genes, because the transcription time of each gene may be different from each other.

Our findings reinforce the immunomodulatory effect of bLf already described in the literature and fill the gap in the knowledge about its regulatory effect on the immune response during SARS-CoV-2 infection. Taken together, the results presented here indicate that bLf affects the amount of circulating immune cells in the late stage of infection, whereas, in acute COVID-19, the bLf induces pro-inflammatory cytokines, suggesting that bLf can be explored considering the immunomodulatory effect under evaluation of the time of infection, as an important supplementation that supports immune in the therapy of SARS-CoV-2 infection, promoting immunological homeostasis. Thus, the bLf effects can be further explored in future clinical trials taking into consideration the time of infection, clinical conditions, and in synergism of other drugs that help clear to clear the infection and minimize several damages.

## Data Availability

The datasets presented in this study can be found in online repositories. The names of the repository/repositories and accession number(s) can be found below: Zenodo, https://doi.org/10.5281/zenodo.13845744.
