# Supplementary material for: Immunomodulatory effect of bovine lactoferrin during SARS-CoV-2 infection
Source: Front Immunol. 2024 Oct 17;15:1456634. doi: 10.3389/fimmu.2024.1456634 (PMC11524939; doi:10.3389/fimmu.2024.1456634)
Supplement: Supplementary file 1 [file DataSheet1.pdf]

Supplementary Material

Supplemantary Figures and Tables

Supplementary Table 1. Primers used in gene expression method for *in vitro* assays.

Gene	Sense	Antisense
ATP6V0E1	GTGCTCAGTCTTTGAGGTCAC	AAGTGGTCTGGTTTGGAGGT
CARD9	ACCAGGAGCAGGTTTTGC	GCCATCCTTTCTGCATCTTC
CCL2	ACCGAGAGGCTGAGACTAAC	GCATTGATTGCATCTGGCTGA
CCL5	GGGTGACAAAGACGACTGCT	CCTGCTGCTTTGCCTACATT
CLEC5A	GGCAATGTTACCAATCAGAATCA	TCATTTGGCATTCTTCTCACAG
CXCL10	CCAATTTTGTCCACGTGTTG	TTCTTGATGGCCTTCGATTC
IFIT1	TTACAGCAACCATGAGTACAA	TGCTCCAGACTATCCTTGAC
IFNA1	GCCATCTCTGTCCTCCATGA	TGGTAGAGTTCGGTGCAGAAT
IFNB1	CATTACCTGAAGGCCAAGGA	CAGCATCTGCTGGTTGAAGA
IFNG	TGACCAGAGCATCCAAAAGA	ATTGCTTTGCGTTGGACATT
IL10	ATCGATGACAGCGCCGTAG	GATGCCCCAAGCTGAGAAC
IL12	TTTCTTTTCTCTCTTGCTCTTGC	GTGGAGGTCAGCTGGGAGTA
IL18	TTTAAGGAAATGAATCCTCCTGAT	TTCAAATTGCATCTTATTATCATGTC
IL1B	GGACAGGATATGGAGCAACAA	TCTTTCAACACGCAGGACAG
IL2	AGCTCAAAGCAAAAACTTTCAC	TGTTGTTTCAGATCCCTTTAGTTC
IL6	GCATCTAGATTCTTTGCCTTTTT	GAAAATCATCACTGGTCTTTTGG
IRF3	TCGAGGTGACAGCCTTCTAC	GCCTCACGTAGCTCATCACT
IRF7	TGATGCTGCGGGATAACTC	CCTGTGGTGGTGGGACAG
ISG15	GAGAGGCAGCGAACTCATCT	CCAGCATCTTCACCGTCAG
NCF4	TGGAGGAAGTGAGAGGTGAA	TGTTCAAAGTCACTCTCGGC
NFKB	CGGTGACAGGAGACGTGAA	ACCCAAGCGGTCCAGAA
NQO2	AACTCCTAGAGCGGTCCTTG	GAGTCCAGCAATCTTCCGAC
NRLP3	GCTTCAGGTGTTGGAATTAGACA	GTCGCCCAGGTCATTGTT
SOD2	CAGCGGTAGCACCAGCACTA	GAGCCCAGATACCCCAAAAC
STAT1	AACCGCATGGAAGTCAGGTT	ATGGGCTTCATCAGCAAGGA
STAT2	CCAGATTTGCCCTGTGATCT	AAACCTCATCCACGGTGTTC
TGFB	GACCTTGCTGTACTGCGTGT	AAGGACCTCGGCTGGAAGT
TLR3	TGCAAAAGATTCAAGGTACATCA	CCAGTTCAAGATGCAGTGAGA
TLR4	AGCTTCTTCAGTTTCCCAGAAC	AAAGGCTCCCAGGGCTAAAC
TLR7	AAAAATGGTGTTTCCAATGTG	GTCTGTGCAGTCCACGATCA
TMEM59	ATTTGACTCGGTCTTGGGTG	ACAACTCCTCTTCCTTAGGGT
TNFA	GAGGGTTTGCTACAACATGG	CCCCAGGGACCTCTCTCTA
B2M	GGCTCGCTCGGTGACCCTAGTCTTT	TCTGCAGGCGTATGTATCAGTCTCA
18S	AGGATGAGGTGGAACGTGTG	CTTCACGGAGCTTGTTGTCC
PPIA	TGTTCTTCGACATTGCCGTC	TGTCTGCAAACAGCTCAAAG
GAPDH	AGTGATGGCATGGACTGTGGTCAT	CAACAGCCTCAAGATCATCAGCAA

Supplemantary Table 2. Primers used in gene expression method for *in vivo* assays.

Gene	Sense	Antisense
IL1B	TGCCACCTTTTGACAGTGATG	ATGTGCTGCTGCGAGATTTG
IL6	GTTCTCTGGGAAATCGTGGA	TGTACTCCAGGTAGCTATGG
IL18	TACAAGCATCCAGGCACAGC	GGCAGGAGTCCAGAAAGCAT
IL10	ATTCCCTGGGTGAGAAGCTG	TAGACACCTTGGTCTTGGAGCTTA
CCL2	CACTCACCTGCTGCTACTCA	GAGCTTGGTGACAAAAACTACAGC
IFNB1	AACTCCACCAGCAGACAGTG	TGAGGACATCTCCCACGTCA
TLR4	AACTCAGCAAAGTCCCTGATG	ATTGTTTCAATTTCACACCTGGAT
TLR9	AGAGACCCTGGTGTGGAAC	CTTCGACGGAGAACCATGT
FHT1	CCGAGATGATGTGGCTCTGAA	CAGTCATCACGGTCTGGTTTCTTT
NOX1	AGTTTCTCTCCCGAAGGACCTC	GGTTAACCAGCCAGTTTCCCA
CASP1	TGGTCTTGTGACTTGGAGGA	TGGCTTCTTATTGGCACGAT
GADD45G	AAGTCCTGAATGTGGACCCTG	ATGGATCTGCAGCGCTATGT
CLEC5A	TTTTTCTGCTGTATTTCCCACA	ACGAAGCCATCATTACTTTTGC
B2M	CAAGACCGTCTACTGGGATCG	TTGCTATTTCTTTCTGCGTGC
PPIA	GCTTTTCGCCGCTTGCT	CTCGTCATCGGCCGTGAT
GAPDH	AACTTTGGCATTGTGGAAGG	GGAGACAACCTGGTCCTCAG

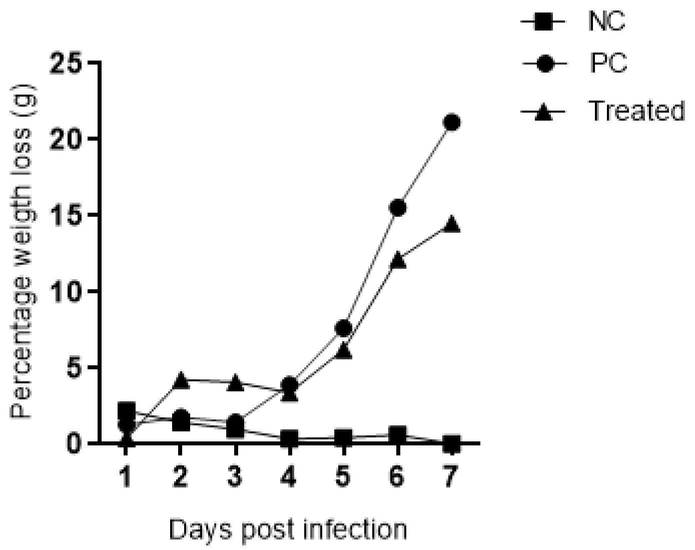

Supplementary Figure 1. Percentage weight loss from k18-ACE2 mice groups infected with SARS-CoV-2 Wuhan strain and treated with bovine lactoferrin (Treated). Lengende: NC= refers to non-infected, non-treated animals; PC= positive control, infected animals; Treated= infected, treated animals.

frontiers

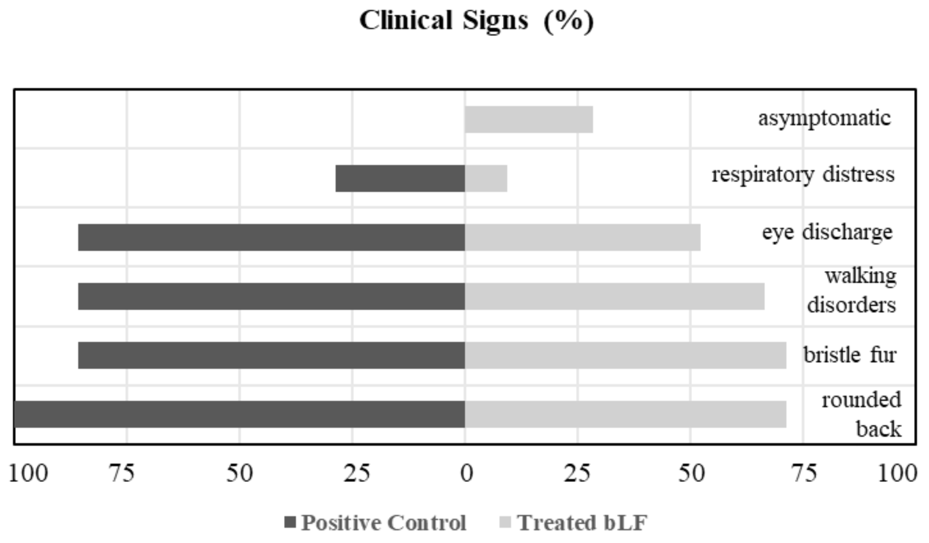

Supplementary Figure 2. The percentage of the clinical sings presented from k18-ACE2 mice groups infected with SARS-CoV-2 Wuhan strain and treated with bLf.
